# Freewheeling sisters cause problems

**DOI:** 10.7554/eLife.13788

**Published:** 2016-02-02

**Authors:** Takashi Akera, Michael A Lampson

**Affiliations:** Department of Biology, University of Pennsylvania, Philadelphia, United States; Department of Biology, University of Pennsylvania, Philadelphia, United Stateslampson@sas.upenn.edu

**Keywords:** meiosis, aneuploidy, kinetochore, human oocytes, chromosome, maternal age effect, Human, Mouse

## Abstract

The factors that lead to errors in chromosome segregation during the production of egg cells in humans are becoming clearer.

**Related research article** Zielinska AP, Holubcova Z, Blayney M, Elder K, Schuh M. 2015. Sister kinetochore splitting and precocious disintegration of bivalents could explain the maternal age effect. *eLife* **4**:e11389. doi: 10.7554/eLife.11389

**Image** Poorly fused kinetochores (indicated by arrows) allow chromosomes to twist before they are separated

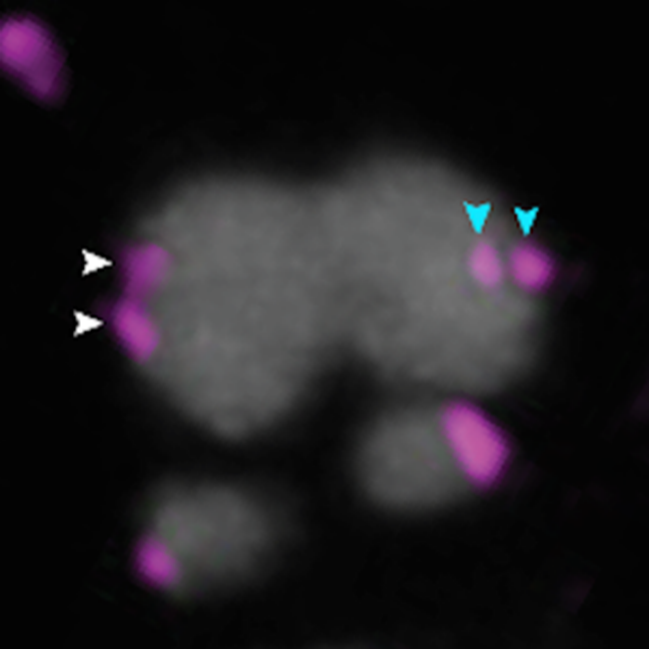

Chromosomes must be accurately segregated during the production of sex cells to ensure that the next generation inherits an intact copy of the genome. However, this process is particularly error-prone in women and gets worse as they get older (Hassold and Hunt, 2001). Errors in chromosome segregation produce egg cells with the wrong number of chromosomes, which can lead to infertility and Down syndrome (Herbert et al., 2015).

New eggs develop from immature egg cells (or oocytes) via meiosis: this process involves the original cell’s DNA being replicated once before it divides twice to produce four new cells, each with half the original number of chromosomes. Most errors in the number of chromosomes in human eggs come from mistakes made when the oocyte divides for the first time in a process commonly called meiosis I. Multiple factors can contribute to these errors, but it is not clear which are most significant in human oocytes (Holubcova et al., 2015; Nagaoka et al., 2012).

Human oocytes start with 23 pairs of homologous chromosomes, which are split during the anaphase stage of meiosis I so that the egg contains one from each pair. There are two main requirements that must be met during meiosis I. First, each pair of homologous chromosomes must be physically connected to form a “bivalent”. Second, the two sister kinetochores on each chromosome must be functionally fused together so that both sisters connect to the same spindle pole (Figure 1). Now, in eLife, Melina Schuh and colleagues at the MRC Laboratory of Molecular Biology, the Max Planck Institute for Biophysical Chemistry and the Bourn Hall Clinic show that these two requirements are both compromised in human oocytes. This provides a plausible mechanism to explain the errors often seen in meiosis I in women (Zielinska et al., 2015).

**Figure 1.**
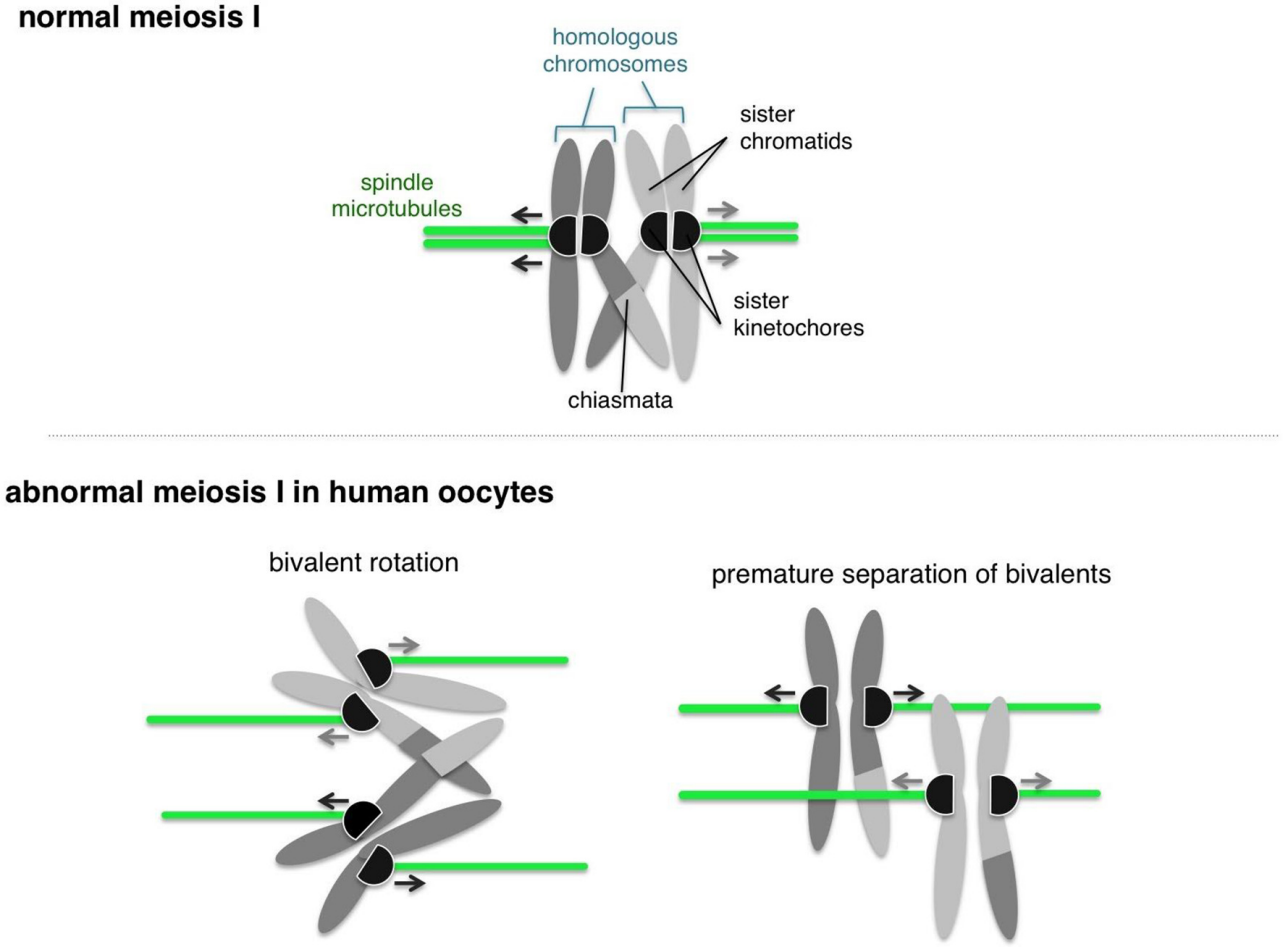
Normal and abnormal meiosis I in human oocytes. Before meiosis, the 46 chromosomes are duplicated such that each consists of two sister chromatids. During meiosis I, the homologous chromosomes then partner up into 23 pairs. A single pair of homologous chromosomes is shown (top), and the two chromosomes are connected via crossovers called “chiasmata”. The kinetochores (shown as black semi-circles) on sister chromatids are fused together and become attached to microtubules (shown in green) emanating from the same pole of the spindle (not shown). This geometry helps the kinetochores of pairs of homologous chromosomes to be captured by microtubules from opposite spindle poles, and pulled to opposite sides of the cell. Zielinska et al. occasionally observed abnormal configurations of chromosomes in human oocytes. Often the sister kinetochores were split and ended up attached to spindle microtubules from different spindle poles (bottom left). This allowed the pair of homologous chromosomes (or "bivalent”) to rotate. Also, the connections between homologous chromosomes were commonly compromised, which sometimes led to premature separation of the bivalent (bottom right).

Schuh and colleagues – who include Agata Zielinska as first author – used optical microscopy to investigate the geometry of kinetochores in oocytes from women of various ages. They observed that sister kinetochores that should have been fused during meiosis I were often disconnected (Figure 1). This splitting of sister kinetochores has typically not been observed in mice and yeast (Kim et al., 2015). Older women also had more split kinetochores than younger women, which is consistent with the findings of previous studies involving mice and humans (Lister et al., 2010; Chiang et al., 2010; Sakakibara et al., 2015).

Zielinska et al. also found that split kinetochores did not attach properly to the spindle that pulls the chromosomes apart during meiosis, which allowed the bivalent to twist or rotate (Figure 1). Rotating bivalents could lead to errors in segregation of the chromosomes, such as replicated copies of each chromosome separating too soon. However, it is still unclear whether these rotated bivalents contribute to cells having the wrong number of chromosomes (a phenomenon known as aneuploidy). An important next step in this research is to investigate how rotated bivalents segregate during anaphase.

A previous study showed that segregation errors in oocytes from older mice occur when a bivalent separates prematurely (Sakakibara et al., 2015). Zielinska et al. show that this also occurs in human oocytes, including oocytes from younger women (Figure 1). This finding may explain why human oocytes are highly error-prone, even at an earlier age, compared to other species (Hassold and Hunt, 2001).

Finally, Zielienska et al. also show that human oocytes proceed to anaphase even if their chromosomes are not aligned correctly. During mitotic cell division, a surveillance system called the spindle assembly checkpoint (SAC) prevents the start of anaphase until all the kinetochores are attached to the spindle: this helps to ensure that the chromosomes are segregated correctly. The work of Zielinska et al. indicates at least two, not mutually exclusive, possibilities for how the SAC works during meiosis in humans. First, it is possible that the SAC does not respond to attachment defects that are associated with the chromosomes not being aligned correctly. Second, the kinetochores may be attached to the spindle, even on the misaligned chromosomes, and therefore do not activate the SAC.

Zielinska et al. suggest that the splitting of sister kinetochores and the premature separation of a bivalent in human oocytes could lead to aneuploidy in the resulting eggs. Complexes containing a cohesion protein called Rec8 are essential for both sister kinetochore fusion and bivalent formation (Watanabe, 2012), and Meikin family proteins work through Rec8 to fuse sister kinetochores (Kim et al., 2015). Thus, it would be interesting to study the Meikin-Rec8 pathway in human oocytes. Rec8 is found along the length of each chromosome (because it connects the homologous chromosomes), as well as near the kinetochore, so examining where Rec8 is localized on the chromosome axis in human oocytes might help us understand why bivalents separate prematurely. Furthermore, the increased separation of sister kinetochores and bivalents that occurs with age may also be explained by defects in Rec8’s function, because previous studies reported that less Rec8 cohesin was associated with chromosomes in older mice (Lister et al., 2010; Chiang et al., 2010).

Last year Schuh and co-workers reported that defects in the formation of the spindle contribute to problems with chromosome segregation in human oocytes (Holubcova et al., 2015). Thus, multiple features of meiosis in human oocytes have been described recently, and further work may identify the major factor, or factors, that contribute to aneuploidy.
